# Plasma-based antigen persistence in the post-acute phase of SARS-CoV-2 infection

**DOI:** 10.1101/2023.10.24.23297114

**Published:** 2023-10-26

**Authors:** Michael J. Peluso, Zoe N. Swank, Sarah A. Goldberg, Scott Lu, Thomas Dalhuisen, Ella Borberg, Yasmeen Senussi, Michael A. Luna, Celina Chang Song, Alexus Clark, Andhy Zamora, Megan Lew, Badri Viswanathan, Beatrice Huang, Khamal Anglin, Rebecca Hoh, Priscila Y. Hsue, Matthew S. Durstenfeld, Matthew A. Spinelli, David V. Glidden, Timothy J. Henrich, J. Daniel Kelly, Steven G. Deeks, David R. Walt, Jeffrey N. Martin

**Affiliations:** 1 Division of HIV, Infectious Diseases, and Global Medicine, University of California, San Francisco, San Francisco, CA, USA; 2 Harvard Medical School, Boston, MA, USA; 3 Department of Pathology, Brigham & Women’s Hospital, Boston, MA, USA; 4 Wyss Institute for Biologically Inspired Engineering, Harvard University, Boston, MA, USA; 5 Department of Epidemiology and Biostatistics, University of California, San Francisco, San Francisco, CA, USA; 6 Division of Cardiology, University of California, San Francisco, San Francisco, CA, USA; 7 Division of Experimental Medicine, University of California, San Francisco, San Francisco, CA, USA

**Keywords:** COVID-19, post-acute sequelae of SARS-CoV-2, Long COVID, viral persistence, antigen

## Abstract

**BACKGROUND:**

Although RNA viruses like SARS-CoV-2 are generally thought to be transient, the persistence of viral components beyond the acute phase can be driven by a variety of virologic and immunologic factors. Recent studies have suggested that SARS-CoV-2 antigens may persist following COVID-19 but were limited by a lack of comparison to a large number of true negative control samples.

**METHODS:**

Using single molecule array (Simoa) assays for SARS-CoV-2 spike, S1, and nucleocapsid antigen in plasma from 171 pandemic-era individuals in the post-acute phase of SARS-CoV-2 infection and 250 pre-pandemic control samples, we compared prevalence of antigen detection. We used logistic regression models and prevalence ratios (PRs) to assess the relationship between demographic and disease factors and antigen persistence.

**RESULTS:**

Compared to the proportion of antigen positivity in the pre-pandemic controls (2%), detection of any SARS-CoV-2 antigen was more frequent across all post-acute COVID-19 time bins (3–6 months: 12.6%, p<0.001; 6–10 months, 10.7%, p=0.0002; 10–14 months, 7.5%, p=0.017). These differences were driven by spike protein for up to 14 months and nucleocapsid in the first 6 months after infection. The co-occurrence of multiple antigens at a single timepoint was uncommon. Hospitalization for acute COVID-19 (versus not hospitalized) and worse self-reported health during acute COVID-19 among those not hospitalized (versus more benign illness) were associated with higher prevalence of post-acute antigen detection (PR 1.86, p=0.03; PR 3.5, p=0.07, respectively) in the pandemic era.

**CONCLUSIONS:**

Our findings provide strong evidence that SARS-CoV-2 antigens can persist beyond the period of acute illness. The observation that more than 10% of plasma samples for over a year following initial SARS-CoV-2 infection contain detectable viral antigen, which are potentially immunogenic, has significant implications given the sheer number of people infected with SARS-CoV-2 to date. More work will be needed to determine whether these antigens have a causal role in post-acute sequelae of SARS-CoV-2 infection (PASC).

## BACKGROUND

Components of certain RNA viruses may persist for weeks or months following acute infection.^1^ Although such pathogens are generally thought to be transient, the persistence of viral components beyond the acute phase can be driven by a variety of virologic and immunologic factors. In some cases, persistence of active virus or viral antigens is associated with post-acute illness.^2^

Early in the pandemic, prolonged nasopharyngeal and/or gastrointestinal shedding of presumably non-infectious virus was observed in some individuals for 90 days or more, often despite clinical recovery.^3,4^ More recently, evidence has suggested that SARS-CoV-2 protein and/or RNA can be detected beyond the acute phase of illness in a subset of individuals.^5^ For example, viral RNA or protein persistence has since been identified in a variety of tissues in gut biopsy and autopsy studies for up to 2 years post-infection, although the clinical significance is unclear.^6–9^ Recent studies assessing protein and RNA in plasma reported antigen detection in small cohorts of individuals post-COVID,^10,11^ but their conclusions were limited by sporadic detection at or near the assay quantification limit and a lack of comparison to a large number of true negative control samples.

In this study, we first used a pre-pandemic sample cohort to determine the true false positive prevalence of the assay. We then measured the presence of SARS-CoV-2 antigen in plasma from a large, well-characterized cohort of persons in the post-acute phase of COVID-19 to determine whether antigen could be detected. We compared the prevalence after COVID-19 to that measured using plasma collected from true negative control participants prior to the emergence of SARS-CoV-2 infection. We hypothesized that the prevalence of antigen in plasma during the post-acute phase of COVID-19 would be significantly higher than in control specimens and would correlate with parameters such as illness severity during the acute phase of infection as well as clinical characteristics such as HIV infection status.

## METHODS

### Overall Design

In cross-sectional analyses, we compared participants in the post-acute phase of SARS-CoV-2 infection to persons studied prior to the COVID-19 pandemic for the presence of three different SARS-CoV-2 antigens in their plasma. Among the persons in the post-acute phase of SARS-CoV-2 infection, we also evaluated a variety of sociodemographic and clinical factors related to the acute phase of COVID-19 for their influence on SARS-CoV-2 antigen detection in the post-acute phase.

### Participants

We studied two groups of participants. The first (hereafter known as pandemic-era) were participants in the University of California, San-Francisco (UCSF)-based Long-term Impact of Infection with Novel Coronavirus (LIINC) study (NCT04362150). Selection of participants into LIINC has been described previously.^12^ Briefly, using facility- and community-based advertising, we enrolled (beginning in April 2020) consecutive adult volunteers who had earlier experienced their first episode of acute SARS-CoV-2 infection (confirmed by detection of SARS-CoV-2 RNA or antigen) and who were at least two weeks removed from their onset of COVID-19-related symptoms. Participants were examined at an initial study visit and every four months thereafter. For the present analysis, we sampled participants who had the greatest number of completed study visits (with stored plasma specimens) in the first 1.25 years following COVID-19 onset. The second group of participants (hereafter known as pre-pandemic-era) were from the UCSF-based Study of the Consequences of the Protease Era (SCOPE), an omnibus cohort study begun in 2001 originally focused on investigation of the pathogenesis of HIV infection. It contains participants at variety of stages of HIV infection as well as ambulatory HIV-uninfected comparators, all of whom were volunteers from the community. For the present analysis, we randomly selected, among participants with stored plasma specimens prior to December 2019, four HIV-uninfected participants to every one HIV-infected participant, attempting to match the age and race/ethnicity distribution of the pandemic-era group. All participants provided written informed consent.

### Measurements

#### Questionnaire-based.

In both groups, interviewer-administered questionnaires collected data on sociodemographic and economic characteristics. In the pandemic-era group, we also inquired about details concerning the acute phase of SARS-CoV-2 infection, including symptoms experienced in the first 3 weeks, self-reported worst perception of overall health on a 0 to 100 scale in the first 3 weeks, and whether hospitalization for COVID-19 occurred. The pandemic-era group also had all SARS-CoV-2 vaccinations recorded as well as any additional SARS-Co-V-2 infections since the initial infection.

#### Laboratory-based measurements.

Peripheral blood was collected in EDTA-coated tubes and plasma stored at −80° C using similar procedures in both the pre-pandemic era and pandemic-era groups. Using once-thawed plasma, we employed single molecule array (Simoa) assays to measure SARS-CoV-2 antigens from spike, S1, and nucleocapsid proteins; detailed methods have been described elsewhere.^10,13^ Briefly, plasma samples were centrifuged at 2000 × g for 10 minutes at 4 °C and treated with 5 mM dithiothreitol (Pierce^™^ No-Weigh^™^ Format, Thermo Fisher Scientific) and protease inhibitors (Halt^™^ Protease Inhibitor Cocktail, Thermo Fisher Scientific) for 15 minutes at 37° C. Each plasma sample was diluted 8-fold in a 96-well plate with Sample Diluent Buffer (Quanterix) and analyzed automatically with a three-step format on a HD-X Analyzer (Quanterix). In the first step, the plasma samples are incubated with antibody-coated magnetic beads. Assays for S1, spike, and nucleocapsid were performed separately, using antibodies against S1 (40150-D006, Sino Biological), S2 (MA5–35946, Invitrogen), and nucleocapsid (40143-R004, Sino Biological) conjugated to carboxylated magnetic beads (Quanterix). In the second step, the beads are resuspended in a solution of biotinylated detector antibodies. The same detector antibody against S1 is used for the S1 and spike assays (LT-1900, Leinco) and another antibody against nucleocapsid is used for the nucleocapsid assay (40143-R040, Sino Biological). In the third step, the beads are incubated in a solution of streptavidin conjugated β-galactosidase and lastly resuspended in a solution of resorufin β-D-galactopyranoside and loaded into a microwell array. The array is then sealed with oil and imaged. Average enzyme per bead (AEB) values are calculated by the HD-X Analyzer software thereafter and converted to concentration values based on a calibration curve fit with a four-parameter logistic regression. Separately, the limit of detection (LOD) is calculated as the background AEB plus three times the standard deviation and converted to a concentration. Only values above the LOD are reported and values below the LOD are set to the LOD.

### Statistical analysis

Up to 7 plasma timepoints (median 4, IQR 3–5) were assayed for SARS-CoV-2 antigens in the pandemic era group and one timepoint in the pre-pandemic group. In each plasma specimen tested, antigen detection was defined in four ways: a) presence or absence on each of three individual antigen assays; and b) presence of at least one of the three antigens (vs absence on all three). When comparing antigen prevalence in the pandemic-era group to the pre-pandemic era group, we defined three periods of time for the pandemic-era group: 3.0–6.0 months, 6.1–10.0 months, and 10.1–14 months post-onset of COVID-19 symptoms. If there was more than one time point per person in a given time period, we chose the timepoint closest to the midpoint of the period. Comparison between groups were expressed with prevalence ratios and differences. All analyses were performed using Stata version 17.0 (StataCorp, College Station, Texas).

## RESULTS

### Study participants

We studied 171 pandemic-era participants over 660 timepoints between 0.9 and 15.4 months following initial SARS-CoV-2 symptom onset (Table 1). The pandemic-era group was 50% female, and median age was 46 years (IQR 37–57). The parent study was deliberatively enriched for people with HIV (PWH); as a result, this comorbidity was relatively common in the study population (n=25, 15%). The pre-pandemic-era group (n=250) was similar in comparison, although included a lower proportion of women (22%).

### Antigen detection

At least one SARS-CoV-2 antigen was detected in 5/250 (2%) pre-pandemic samples. In the pre-pandemic samples, spike was detected in 3/250 (1.2%) cases, S1 in 3/250 (1.2%) cases, and N in 2/250 (0.8%) cases. These values represent prevalence of false positivity. Of those with false positive detectable antigen, positive Spike values were: 83.46 pg/mL, 609.96 pg/mL, and 646.02 pg/mL; positive S1 values were: 34.67 pg/mL, 115.89 pg/mL, and 285.31 pg/mL; positive N values were: 649.28 pg/mL and 5716.32 pg/mL.

**Figure 1.**
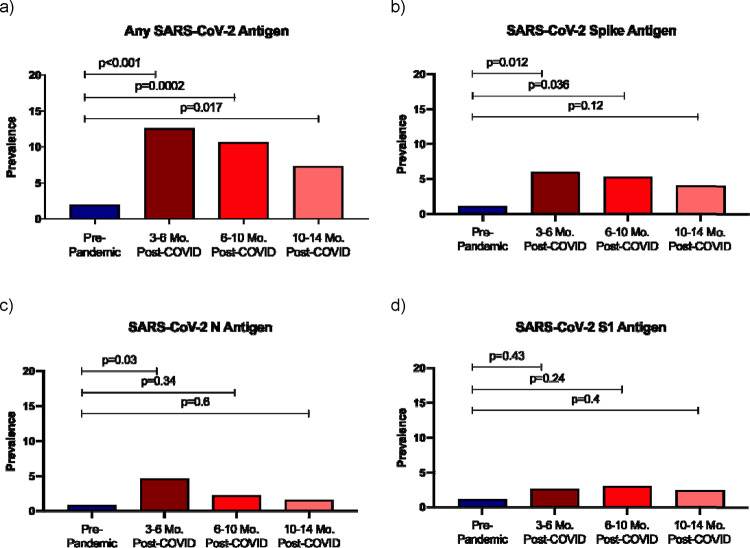
(a) Prevalence of SARS-CoV-2 antigen in plasma in the post-acute phase of COVID-19 in comparison to prevalence of false positivity in pre-pandemic control plasma. (b) Spike antigen prevalence. (c) Nucleocapsid antigen presence. (d) S1 presence. P-values represent Chi-square and Fisher’s 2-sided exact test as appropriate.

Compared to the proportion of antigen positivity in the pre-pandemic group, detection of any SARS-CoV-2 antigen was more frequent across all pandemic-era time bins (Figure 1a; Supplemental Table 1). These differences were driven by spike protein for up to 14 months and nucleocapsid in the first 6 months after infection (Figure 1b–d).

**Figure 2.**
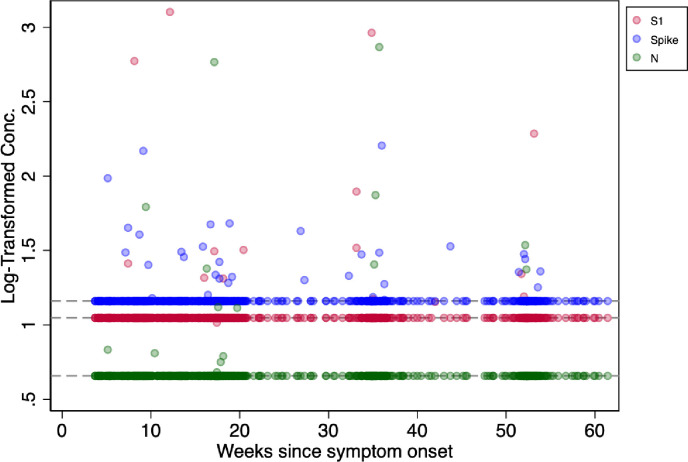
Plasma antigen measurements. Dashed lines indicate limit of quantification for each analyte. Y-axis refers to log-transformed concentration, units are pg/mL.

Of the 660 pandemic-era timepoints studied, 61 (9.2%) timepoints representing 25% of participants had one or more detectable SARS-CoV-2 antigens (Figure 2). The most commonly detected antigen was Spike (n=33, 5.0%), followed by S1 (n=15, 2.3%) and N (n=15, 2.3%). In most cases (59/61, 96.7%) only a single antigen was detected. The co-occurrence of multiple antigens at a single timepoint was uncommon. In 2/61 cases (4.9%), two antigens were simultaneously detected (one case was positive for S1 and N; a second case was positive for Spike and N). In no case were all three antigens detected simultaneously. Of those with detectable antigen, the median Spike concentration was 27.7 pg/mL (IQR 20.5–33.7), median S1 concentration was 31.2 pg/mL (IQR 20.5–193.0), and median N was 23.6 pgmL (IQR 6.46–62.0).

**Figure 3.**
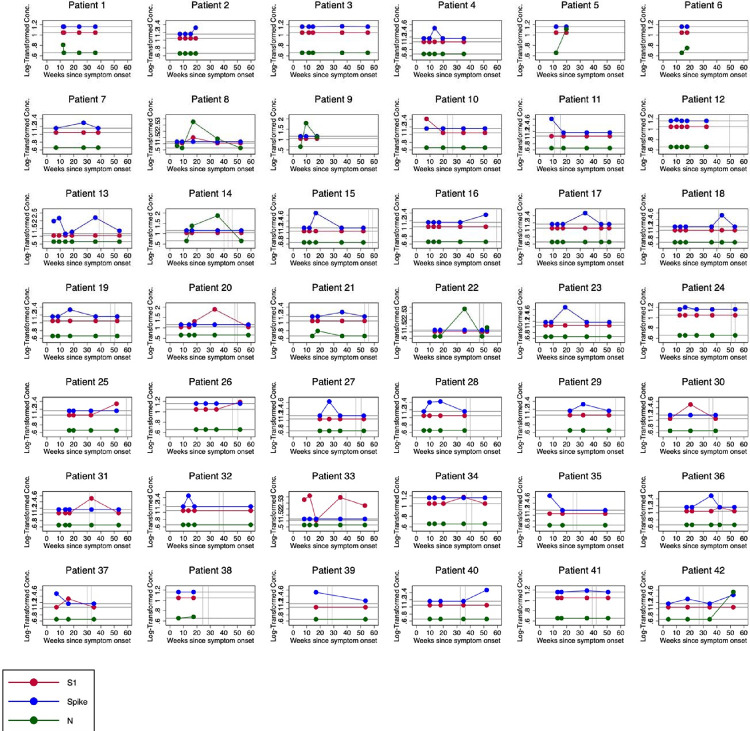
Antigen measurements over time in pandemic-era participants with at least one positive antigen. Blue indicates spike, green nucleocapsid, and red S1. Horizontal dotted lines represent the assay limit of detection for each antigen. Vertical dotted lines indicate timing of any SARS-CoV-2 vaccine relative to antigen.

It was also uncommon for individuals to demonstrate the presence of antigen at multiple timepoints. Of 159 participants who had multiple timepoints studied, 29 (18.2%) had antigen detected at a single post-acute timepoint, 10 (6.3%) had antigen detected at two post-acute timepoints, and one (0.63%) had antigen detected at three, four, and five post-acute timepoints, respectively (Figure 3). Most timepoints at which antigen was detected (51/61, 84%) occurred before the participant had ever received a SARS-CoV-2 vaccine (Figure 3). There were five instances in which antigen was detected within three weeks of a SARS-CoV-2 vaccine dose (three for S1, one for Spike, and one for N).

### Determinants of antigen positivity among pandemic-era participants

Finally, we examined determinants of antigen detection among the pandemic-era participants. We found no strong evidence of an association between age, sex, race/ethnicity, HIV status, or body mass index (BMI) with antigen positivity. In contrast, we found strong evidence for an influence of severity of the acute phase of infection. As compared to those not hospitalized, participants who required hospitalization for acute COVID-19 were nearly twice as likely to have antigen detected (prevalence ratio [PR] 1.86, p=0.03). Among those not hospitalized for COVID-19, those with the worst self-report of acute illness were over 3 times as likely to have antigen detected as compared to those with the most benign self-reports (PR 3.5, p=0.07), an absolute difference of nearly 30%.

## DISCUSSION

In comparison to pre-pandemic samples, we identified increased prevalence of SARS-CoV-2 antigen detection for up to 14 months following confirmed SARS-CoV-2 infection. This observation builds upon earlier work in smaller cohorts^10,11^ and provides strong evidence that SARS-CoV-2 should be added to the list of RNA viruses whose components may persist beyond the period of acute illness.^10^ Although a definitive link between antigen persistence and post-acute sequelae (e.g., Long COVID symptoms, cardiovascular disease, stroke, blood clots, etc.) has yet to be confirmed, the observation that more than 10% of plasma samples for over a year following initial SARS-CoV-2 infection contain detectable viral antigens, which are potentially immunogenic,^14–16^ has significant implications given the sheer number of people infected with SARS-CoV-2 to date.

Our study addresses two key criticisms of prior work evaluating SARS-CoV-2 antigen persistence.^5^ First, our direct comparison with SARS-CoV-2 antigen signal measured in pre-pandemic control samples mitigates the concern that antigen detection in the post-acute phase can be entirely explained by false-positive signal. Second, by including many specimens banked prior to the receipt of any SARS-CoV-2 vaccine, we were able to determine that most antigen detection occurred in these pre-vaccine samples, suggesting that this finding is driven by SARS-CoV-2 infection and not prior vaccination (although one would not expect N protein to be present in relation to a SARS-CoV-2 vaccine).

The proportion of antigen positivity was lower in our study than in a prior report using this assay.^10^ The lower prevalence detected in our study may be because we enrolled individuals in the post-acute phase regardless of illness severity or the presence of post-acute symptoms. In contrast, prior reports studied those seeking clinical care in Long COVID clinics, many of whom were highly symptomatic. Further work is needed to assess whether antigen persistence is a driver of the post-acute sequelae of SARS-CoV-2 infection, or whether it is simply a remnant of prior infection with little or no consequences.

As in prior reports, we found antigen detection to be sporadic in individuals in whom it was present. It is possible that small variations over time could result in periods during which antigen is less detectable using current assays. Alternatively, antigen release from tissue reservoirs may in fact be intermittent, driven by host factors that have not yet been determined but might include differential release from tissues based on other factors such as the timing of a meal, hormonal factors, etc. The finding of an association between the severity of the acute phase illness and antigen detection in the post-acute phase also suggests that the burden of infectious antigen established early in the disease course may determine the likelihood of antigen persistence. This might offer one explanation for the protective effect of SARS-CoV-2 vaccination or antiviral therapy.^17–20^ This finding also merits further investigation.

This study adds substantially to prior work that has challenged the framework that SARS-CoV-2 infection is transient in all cases.^5^ The finding that the virus can evolve over months in immunocompromised individuals provides proof that persistent infection is possible.^8^ Among immunocompetent individuals, evolving B cell immunity provided early albeit indirect evidence that SARS-CoV-2 infection can be sustained.^6^ More recently, an autopsy study showed widespread sub-genomic RNA in tissues of decedents up to 6 months post-COVID,^7^ and we have reported SARS-CoV-2 spike RNA detection in gut biopsies for up to 2.5 years post-COVID.^9^ SARS-CoV-2 antigens, particularly spike protein and its components, are known to be highly immunogenic.^14–16^ These antigens have been shown to induce inflammatory cytokine responses as well as dysregulation of fibrinogen, complement proteins, and endothelial cells. Thus, the consistent identification of post-acute antigen in a subset of individuals following SARS-CoV-2 infection provides additional rationale for interventions meant to reduce or eliminate these antigens, either with antiviral drugs (if viral replication is present) or other drugs that could neutralize protein, clear infected cells, and/or modulate the immune response to these antigens (e.g., monoclonal antibodies). Such studies are now underway (NCT05877507, NCT05595369, NCT05576662, NCT05668091).

The strength of our analysis is the comparison to definitively negative pre-pandemic control specimens, demonstrating long-term antigen persistence in a small but significant proportion of individuals following COVID-19. However, the study has several limitations. The cohort was a convenience sample and not representative of all individuals experiencing SARS-CoV-2 infection during the period of interest. We studied samples preceding the emergence of the Omicron strains, and further work will be needed to assess the prevalence of antigen detection following infection with circulating SARS-CoV-2 variants. While our participants did not have known or clinically suspected reinfections prior to antigen detection, they were not systematically assessed for asymptomatic SARS-CoV-2 infection at each time point. Finally, even though the Simoa is highly sensitive, it is possible that more sensitive assays will be needed, particularly as the source of persistent SARS-CoV-2 antigen might not result in the production of a readily detectable systemic infection and many detected values were near the assay limit. Our team is developing such an assay (MOSAIC).^21^

In summary, we described post-acute antigen detection in a cohort of 171 individuals in the post-acute phase following SARS-CoV-2 infection. In comparison to pre-pandemic samples, we demonstrated an increased prevalence of SARS-CoV-2 antigen detection for over 1 year following initial infection. While the overall prevalence of antigen detection was lower than in prior work comprising a different study population, this finding has major implications given the scale of the pandemic and the knowledge that SARS-CoV-2 spike protein is highly immunogenic. Taken together, these results provide strong evidence that SARS-CoV-2 antigen persists in the post-acute phase in some individuals. However, determination of whether antigen persistence is causally related to symptoms will require larger studies of carefully characterized samples, better delineating the test characteristics of these assays, and the use of experimental medicine to probe the biology of this mechanistic pathway.

## Supplementary Material

Supplement 1

## Figures and Tables

**Table 1.** Characteristics of study participants.

Characteristic	Pandemic Era (n = 171)	Pre-pandemic Era (n = 250)

**Age, years**	46	48
	(37–57)^a^	(36–58)^a^
	(19–85)^b^	(22–83)^b^
**Female Birth Sex**	86 (50%)	56 (22%)
**Sexual Orientation** ^c^		
Asexual	1 (0.6%)	0 (0.0%)
Bisexual	1 (0.6%)	24 (9.6%)
Gay/lesbian	34 (20%)	98 (39%)
Straight/heterosexual	110 (64%)	113 (45%)
Other	1 (0.6%)	0 (0%)
**Race/Ethnicity** ^c^		
Hispanic/Latino	47 (28%)	41 (16%)
White	92 (54%)	104 (42%)
Black/African American	8 (4.7%)	76 (30%)
Asian	17 (9.9%)	29 (12%)
Pacific Islander/Native Hawaiian	3 (1.8%)	0 (0.0%)
**Education** ^c^		
Any high school or less	33 (19%)	79 (32%)
Any college	69 (40%)	117 (47%)
Any graduate school	69 (40%)	52 (21%)
**Income** ^c^		
$30,000 or less	24 (14%)	115 (46%)
$30,001 to $70,000	24 (14%)	36 (14%)
More than $70,000	99 (58%)	40 (16%)
**Body Mass Index** ^c^		
Less than 18.5 kg/m^2^	2 (1.2%)	5 (2.0%)
18.5 kg/m^2^ to 24.9 kg/m^2^	58 (34%)	116 (46%)
25.0 kg/m^2^ to 29.9 kg/m^2^	49 (29%)	68 (27%)
More than 30.0 kg/m^2^	61 (36%)	50 (20%)
**HIV Seropositive** ^c^	25 (15%)	50 (20%)
**Hospitalized During Acute COVID Infection** ^c^	33 (19%)	N/A
**Symptom Count During Acute COVID Infection**	9	N/A
	(6–12)^a^	
	(0–25)^b^	
**Health Score at Worst Point in COVID Illness** ^c^	45	N/A
	(25–60)^a^	
	(0–100)^b^	
**Statement of Health** ^c^		
Excellent	N/A	62 (25%)
Very Good	N/A	82 (33%)
Good	N/A	65 (26%)
Fair	N/A	26 (10%)
Poor	N/A	3 (1.2%)

a Median (interquartile range)

b Absolute range

c Missing and nonresponse. Sexual orientation: 35 missing, 3 questioning/unsure, 1 prefer not to answer; race/ethnicity: 4 missing; education: 2 missing; income: 3 missing, 80 prefer not to answer; BMI: 12 missing; hospitalization: 1 missing; health score: 82 missing; statement of health: 12 missing

**Table 2.** Association between sociodemographic and clinical characteristics and antigen positivity.

Characteristic	Prevalence	Prevalence Ratio (95% CI)	Prevalence Difference (95% CI)	p-value

**Age, years**				
Less than 40	0.26	Ref	Ref	
40–65	0.21	0.81 (0.45 to 1.44)	−0.05 (−0.19 to 0.09)	0.47
65 or more	0.40	1.52 (0.71 to 3.25)	0.14 (−0.14 to 0.41)	0.31
**Sex**				
Female	0.20	Ref	Ref	
Male	0.29	1.49 (0.87 to 2.55)	0.10 (−0.03 to 0.23)	0.15
**Race/ethnicity**				
White	0.23	Ref	Ref	
Hispanic/Latino	0.32	1.40 (0.80 to 2.46)	0.09 (−0.07 to 0.25)	0.25
Black/African American	0.25	1.10 (0.31 to 3.87)	0.02 (−0.29 to 0.33)	0.89
Asian	0.18	0.77 (0.26 to 2.32)	−0.05 (−0.25 to 0.15)	0.64
Pacific Islander/Native Hawaiian	0.33	1.46 (0.28 to 7.59)	0.11 (−0.44 to 0.65)	0.68
**HIV**				
No	0.25	Ref	Ref	
Yes	0.24	0.97 (0.46 to 2.07)	−0.01 (−0.19 to 0.18)	0.94
**Body Mass Index** ^a^				
Less than 18.5 kg/m^2^	-	-	-	-
18.5 kg/m^2^ to 24.9 kg/m^2^	0.21	Ref	Ref	
25.0 kg/m^2^ to 29.9 kg/m^2^	0.29	1.38 (0.70 to 2.71)	0.08 (−0.09 to 0.24)	0.35
More than 30.0 kg/m^2^	0.26	1.27 (0.66 to 2.45)	0.06 (−0.10 to 0.21)	0.48
**Hospitalized During Acute COVID Infection**				
No	0.21	Ref	Ref	
Yes	0.39	1.86 (1.09 to 3.17)	0.18 (0.00 to 0.36)	0.03
**Symptom Count During Acute COVID Infection** ^b,c^				
0–5 symptoms	0.23	Ref	Ref	
6–8 symptoms	0.19	0.86 (0.34 to 2.19)	−0.03 (−0.23 to 0.17)	0.75
9–11 symptoms	0.10	0.46 (0.13 to 1.61)	−0.12 (−0.31 to 0.06)	0.22
12+ symptoms	0.29	1.27 (0.56 to 2.85)	0.06 (−0.14 to 0.26)	0.57
**Health Score at Worst Point in COVID Infection** ^b,d^				
>60	0.12	Ref	Ref	
50–60	0.13	1.11 (0.21 to 5.99)	0.01 (−0.19 to 0.22)	0.90
30–49	0.20	1.70 (0.35 to 8.25)	0.08 (−0.15 to 0.32)	0.51
<30	0.41	3.50 (0.84 to 14.6)	0.29 (0.01 to 0.58)	0.07

a Individuals whose BMI was <18.5 kg/m^2^ (N=2) were omitted from the analyses due to collinearity

b Analyses only conducted amongst participants who were not hospitalized during their acute COVID infection

c N= 138

d N= 77
