# Supplementary material for: Plasma-based antigen persistence in the post-acute phase of SARS-CoV-2 infection
Source: medRxiv. 2023 Oct 26:2023.10.24.23297114. Preprint. [Version 2] doi: 10.1101/2023.10.24.23297114 (PMC10635183; doi:10.1101/2023.10.24.23297114)
Supplement: Supplementary file 1 [file NIHPP2023.10.24.23297114v2-supplement-1.pdf]

medRxiv preprint doi: https://doi.org/10.1101/2023.10.24.23297114; this version posted October 26, 2023. The copyright holder for this preprint (which was not certified by peer review) is the author/funder, who has granted medRxiv a license to display the preprint in perpetuity.
It is made available under a CC-BY 4.0 International license .

Supplemental Table 1.

Any SARS-CoV-2 Antigen

Group	No. of participants	No. of antigen positive (%)	Prevalence difference; 95% CI; p value
Pre-pandemic	250	5 (2.00%)	Reference
3 to 6.0 months after infection	151	19 (12.58%)	0.1058 (0.0502 to 0.1615; p-value < 0.001)
6 to 10.0 months	131	14 (10.69%)	0.0869 (0.0312 to 0.1425; p-value = 0.0002)
10 to 14 months*	122	9 (7.38%)	0.0538 (0.0042 to 0.0103; p-value = 0.017)

*Fisher's 2-sided exact-test

Spike*

Group	No. of participants	No. of antigen positive (%)	Prevalence difference; 95% CI; p value
Pre-pandemic	250	3 (1.20%)	Reference
3 to 6.0 months after infection	151	9 (5.96%)	0.0476 (0.0075 to 0.0877; p-value = 0.012)
6 to 10.0 months	131	7 (5.34%)	0.0414 (0.0006 to 0.0822; p-value = 0.036)
10 to 14 months	122	5 (4.10%)	0.0289 (-0.0087 to 0.0667; p-value = 0.12)

*Fisher's 2-sided exact-test

N*

Group	No. of participants	No. of antigen positive (%)	Prevalence difference; 95% CI; p value
Pre-pandemic	250	2 (0.80%)	Reference
3 to 6.0 months after infection	151	7 (4.64%)	0.0384 (0.0031 to 0.0737; p-value = 0.030)
6 to 10.0 months	131	3 (2.29%)	0.0149 (-0.0130 to 0.0428; p-value = 0.34)
10 to 14 months	122	2 (1.64%)	0.0084 (-0.0167 to 0.0335; p-value = 0.60)

*Fisher's 2-sided exact-test

S1*

medRxiv preprint doi: https://doi.org/10.1101/2023.10.24.23297114; this version posted October 26, 2023. The copyright holder for this preprint (which was not certified by peer review) is the author/funder, who has granted medRxiv a license to display the preprint in perpetuity.
It is made available under a CC-BY 4.0 International license .

Group	No. of participants	No. of antigen positive (%)	Prevalence difference; 95% CI; p value
Pre-pandemic	250	3 (1.20%)	Reference
3 to 6.0 months after infection	151	4 (2.65%)	0.0145 (-0.0145 to 0.0434; p-value = 0.43)
6 to 10.0 months	131	4 (3.05%)	0.0185 (-0.0139 to 0.0509; p-value = 0.24)
10 to 14 months	122	3 (2.46%)	0.0126 (-0.0180 to 0.0432; p-value = 0.40)

*Fisher's 2-sided exact-test

medRxiv preprint doi: https://doi.org/10.1101/2023.10.24.23297114; this version posted October 26, 2023. The copyright holder for this preprint (which was not certified by peer review) is the author/funder, who has granted medRxiv a license to display the preprint in perpetuity.
It is made available under a CC-BY 4.0 International license .

Supplemental Table 2a. Association between sociodemographic characteristics and ever S1 antigen positivity

Characteristic	Prevalence	Prevalence Ratio (95% CI)	Prevalence Difference (95% CI)	p-value
**Age, years**				
Less than 40	0.05	Ref	Ref	
40-65	0.04	0.77 (0.18 to 3.32)	-0.01 (-0.08 to 0.06)	0.72
65 or more	0.27	5.07 (1.26 to 20.3)	0.21 (-0.02 to 0.45)	0.02
**Sex**				
Female	0.05	Ref	Ref	
Male	0.08	1.77 (0.54 to 5.85)	0.04 (-0.04 to 0.11)	0.35
**Race/ethnicity**[a]				
White	0.08	Ref	Ref	
Hispanic/Latino	0.06	0.84 (0.23 to 3.11)	-0.01 (-0.10 to 0.08)	0.79
Black/African American	-	-	-	-
Asian	0.06	0.77 (0.10 to 5.93)	-0.02 (-0.14 to 0.11)	0.80
Pacific Islander/Native Hawaiian	-	-	-	-
**HIV**				
No	0.07	Ref	Ref	
Yes	0.04	0.58 (0.08 to 4.39)	-0.03 (-0.12 to 0.06)	0.60
**Body Mass Index**[a]				
Less than 18.5 kg/m^2	-	-	-	-
18.5 kg/m^2 to 24.9 kg/m^2	0.12	Ref	Ref	
25.0 kg/m^2 to 29.9 kg/m^2	0.04	0.34 (0.07 to 1.56)	-0.08 (-0.18 to 0.02)	0.16
More than 30.0 kg/m^2	0.03	0.27 (0.06 to 1.26)	-0.09 (-0.18 to 0.01)	0.09
**Hospitalized During Acute COVID Infection**				
No	0.06	Ref	Ref	
Yes	0.09	1.56 (0.43 to 5.57)	0.03 (-0.07 to 0.14)	0.50
**Symptom Count During Acute COVID Infection**[b]				
0-5 symptoms	0.10	Ref	Ref	
6-8 symptoms	0.06	0.57 (0.10 to 3.24)	-0.04 (-0.17 to 0.09)	0.53
9-11 symptoms	0.03	0.36 (0.04 to 3.26)	-0.06 (-0.19 to 0.06)	0.36
12+ symptoms	0.05	0.49 (0.09 to 2.79)	-0.05 (-0.17 to 0.07)	0.42
**Health Score at Worst Point in COVID Illness**[a,b]				
>60	0.06	Ref	Ref	
50-60	0.09	1.48 (0.14 to 15.3)	0.03 (-0.13 to 0.19)	0.74
30-49	0.15	2.55 (0.29 to 22.7)	0.09 (-0.10 to 0.29)	0.39
<30	-	-	-	-

[a]Individuals who are Black/African American (N=8) or Pacific Islander/Native Hawaiian (N=3), whose BMI was <18.5 kg/m^2 (N=2), or whose health score was <30 (N=17) were omitted from the analyses due to collinearity
[b]Analyses only conducted amongst participants who were not hospitalized during their acute COVID infection
[c]N= 138
[d]N= 60

medRxiv preprint doi: https://doi.org/10.1101/2023.10.24.23297114; this version posted October 26, 2023. The copyright holder for this preprint (which was not certified by peer review) is the author/funder, who has granted medRxiv a license to display the preprint in perpetuity.
It is made available under a CC-BY 4.0 International license .

Supplemental Table 2b. Association between sociodemographic characteristics and ever spike antigen positivity

Characteristic	Prevalence	Prevalence Ratio (95% CI)	Prevalence Difference (95% CI)	p-value
**Age, years**				
Less than 40	0.18	Ref	Ref	
40-65	0.13	0.75 (0.35 to 1.60)	-0.04 (-0.16 to 0.08)	0.46
65 or more	0.20	1.14 (0.36 to 3.64)	0.02 (-0.20 to 0.25)	0.83
**Sex**				
Female	0.13	Ref	Ref	
Male	0.18	1.38 (0.67 to 2.83)	0.05 (-0.06 to 0.16)	0.38
**Race/ethnicity**[a]				
White	0.15	Ref	Ref	
Hispanic/Latino	0.19	1.26 (0.59 to 2.70)	0.04 (-0.10 to 0.17)	0.56
Black/African American	0.25	1.64 (0.45 to 6.01)	0.10 (-0.21 to 0.41)	0.48
Asian	0.06	0.39 (0.05 to 2.77)	-0.09 (-0.23 to 0.04)	0.33
Pacific Islander/Native Hawaiian	-	-	-	-
**HIV**				
No	0.15	Ref	Ref	
Yes	0.16	1.06 (0.40 to 2.83)	0.01 (-0.15 to 0.16)	0.90
**Body Mass Index**[a]				
Less than 18.5 kg/m^2	-	-	-	-
18.5 kg/m^2 to 24.9 kg/m^2	0.09	Ref	Ref	
25.0 kg/m^2 to 29.9 kg/m^2	0.20	2.37 (0.86 to 6.48)	0.12 (-0.02 to 0.25)	0.09
More than 30.0 kg/m^2	0.18	2.09 (0.77 to 5.67)	0.09 (-0.03 to 0.22)	0.14
**Hospitalized During Acute COVID Infection**				
No	0.15	Ref	Ref	
Yes	0.15	0.99 (0.40 to 2.43)	-0.00 (-0.14 to 0.14)	0.98
**Symptom Count During Acute COVID Infection**[b,c]				
0-5 symptoms	0.16	Ref	Ref	
6-8 symptoms	0.14	0.86 (0.27 to 2.71)	-0.02 (-0.19 to 0.15)	0.80
9-11 symptoms	0.07	0.43 (0.09 to 2.05)	-0.09 (-0.25 to 0.07)	0.28
12+ symptoms	0.21	1.33 (0.49 to 3.59)	0.05 (-0.13 to 0.23)	0.57
**Health Score at Worst Point in COVID Illness**[b,d]				
>60	0.12	Ref	Ref	
50-60	0.09	0.74 (0.11 to 4.79)	-0.03 (-0.22 to 0.16)	0.75
30-49	0.05	0.43 (0.04 to 4.35)	-0.07 (-0.25 to 0.11)	0.47
<30	0.35	3.00 (0.70 to 12.9)	0.24 (-0.04 to 0.51)	0.12

[a]Individuals who are Pacific Islander/Native Hawaiian (N=3) or whose BMI was <18.5 kg/m^2 (N=2) were omitted from the analyses due to collinearity
[b]Analyses only conducted amongst participants who were not hospitalized during their acute COVID infection
[c]N= 138
[d]N= 77

medRxiv preprint doi: https://doi.org/10.1101/2023.10.24.23297114; this version posted October 26, 2023. The copyright holder for this preprint (which was not certified by peer review) is the author/funder, who has granted medRxiv a license to display the preprint in perpetuity.
It is made available under a CC-BY 4.0 International license .

Supplemental Table 2c. Association between sociodemographic characteristics and ever N antigen positivity

Characteristic	Prevalence	Prevalence Ratio (95% CI)	Prevalence Difference (95% CI)	p-value
**Age, years**				
Less than 40	0.05	Ref	Ref	
40-65	0.07	1.34 (0.36 to 5.01)	0.02 (-0.06 to 0.10)	0.66
65 or more	-	-	-	-
**Sex**				
Female	0.05	Ref	Ref	
Male	0.07	1.52 (0.44 to 5.21)	0.02 (-0.05 to 0.09)	0.51
**Race/ethnicity**				
White	0.02	Ref	Ref	
Hispanic/Latino	0.11	4.89 (0.98 to 24.4)	0.08 (-0.01 to 0.18)	0.05
Black/African American	0.13	5.75 (0.58 to 57.1)	0.10 (-0.13 to 0.34)	0.15
Asian	0.06	2.71 (0.26 to 28.4)	0.04 (-0.08 to 0.15)	0.41
Pacific Islander/Native Hawaiian	0.33	15.3 (1.85 to 127)	0.31 (-0.22 to 0.85)	0.03
**HIV**				
No	0.05	Ref	Ref	
Yes	0.08	1.46 (0.33 to 6.51)	0.03 (-0.09 to 0.14)	0.62
**Body Mass Index**[a]				
Less than 18.5 kg/m^2	-	-	-	-
18.5 kg/m^2 to 24.9 kg/m^2	0.05	Ref	Ref	
25.0 kg/m^2 to 29.9 kg/m^2	0.04	0.79 (0.14 to 4.56)	-0.01 (-0.09 to 0.07)	0.79
More than 30.0 kg/m^2	0.08	1.58 (0.39 to 6.36)	0.03 (-0.06 to 0.12)	0.51
**Hospitalized During Acute COVID Infection**				
No	0.02	Ref	Ref	
Yes	0.21	9.69 (2.64 to 35.6)	0.19 (0.05 to 0.33)	<0.001
**Symptom Count During Acute COVID Infection**[b]				
0-5 symptoms	-	-	-	-
6-8 symptoms	-	-	-	-
9-11 symptoms	-	-	-	-
12+ symptoms	-	-	-	-
**Health Score at Worst Point in COVID Illness**[b]				
>60	-	-	-	-
50-60	-	-	-	-
30-49	-	-	-	-
<30	-	-	-	-

[a]Individuals who were 65 years or older (N=15) or whose BMI was <18.5 kg/m^2 (N=2) were omitted from the analyses due to collinearity
[b]Analyses were omitted due to complete collinearity
